# Neuropilar Projections of the Anterior Gastric Receptor Neuron in the Stomatogastric Ganglion of the Jonah Crab, *Cancer Borealis*

**DOI:** 10.1371/journal.pone.0079306

**Published:** 2013-12-03

**Authors:** Marie L. Goeritz, Matthew R. Bowers, Brian Slepian, Eve Marder

**Affiliations:** Biology Department and Volen Center, Brandeis University, Waltham, Massachusetts, United States of America; Claremont Colleges, United States of America

## Abstract

Sensory neurons provide important feedback to pattern-generating motor systems. In the crustacean stomatogastric nervous system (STNS), feedback from the anterior gastric receptor (AGR), a muscle receptor neuron, shapes the activity of motor circuits in the stomatogastric ganglion (STG) via polysynaptic pathways involving anterior ganglia. The AGR soma is located in the dorsal ventricular nerve posterior to the STG and it has been thought that its axon passes through the STG without making contacts. Using high-resolution confocal microscopy with dye-filled neurons, we show here that AGR from the crab *Cancer borealis* also has local projections within the STG and that these projections form candidate contact sites with STG motor neurons or with descending input fibers from other ganglia. We develop and exploit a new masking method that allows us to potentially separate presynaptic and postsynaptic staining of synaptic markers. The AGR processes in the STG show diversity in shape, number of branches and branching structure. The number of AGR projections in the STG ranges from one to three simple to multiply branched processes. The projections come in close contact with gastric motor neurons and descending neurons and may also be electrically coupled to other neurons of the STNS. Thus, in addition to well described long-loop pathways, it is possible that AGR is involved in integration and pattern regulation directly in the STG.

## Introduction

Sensory feedback from muscle receptors shapes motor neuron activity in mono- and disynaptic (“short-loop”) stretch or resistance reflexes. In addition, most reflex pathways have polysynaptic (“long-loop”) components that are crucial to adapt reflexes to different behavioral states. In fact, phase-specific sensory feedback to vertebrate and invertebrate motor neurons during rhythmic motor activity can cause reflexes to reverse or become a component of the rhythm-generating networks themselves [1-7]. 

The underlying mechanisms of sensory integration during reflex modification are not fully understood. However, many functional aspects of feedback integration, such as presynaptic inhibition and antidromic spiking, can be linked to the structures of motor or sensory neurons [8]. Examining the morphology of a neuron in detail opens the door to interesting questions. How variable are the structures of identified neurons across individual animals? Which parameters must be preserved to maintain consistent function within a network? These questions are particularly hard to address in large networks in which cell type identification is ambiguous and exacerbated by complicated branching structures.

 There is a large body of evidence for distinct rules that govern aspects of cell morphology during network development. The distribution of attractant and repellent molecules and the respective receptors in the developing spinal cord can determine the position of the soma, axon growth cone guidance, and midline crossing of the axon [9-13]. The shape and position of the same neuron however often vary from animal to animal, as seen in the crustacean stomatogastric ganglion (STG) where only some features of neuron morphology are shared across animals [14-16]. To better understand these aspects of neuron morphology, here we examine a sensory neuron in the STG that is involved in a rich set of reflex pathways but has a relatively simple branching structure.

Rhythmically active networks in the stomatogastric nervous systems (STNS) control movement of the crustacean stomach muscles. During gastric mill activity, protraction and retraction of the stomach teeth is controlled by coordination of the gastric mill network [17]. As in lobsters and crayfish, the Anterior Gastric Receptor (AGR) in *C. borealis* has a bipolar soma that is located in the in the dorsal ventricular nerve (dvn) or, less often, in the posterior part of the STG and projects to the dorsal gastric nerve (dgn) [18]. Bilateral dendrites project into the two gastric mill 1 (*gm1*) muscles where spikes are initiated close to the *gm1* muscles [19]. The AGR axon projects in a long-loop pathway through the STG and makes excitatory and inhibitory connections in the anterior paired commissural ganglia (CoGs) with projection neurons that feed back to the STG motor circuits [17,20-24]. 

The single AGR is a muscle force receptor in the *gm1* muscles. The AGR neuron integrates proprioceptive information from both sides of the animal but also functions similarly to an interneuron by influencing gastric and pyloric network activity, independent of its receptor properties [25]. Multiple dendritic and axonal spike initiation zones respond to different neuromodulators [21,24-27]. Moreover, neuropeptides switch AGR firing between tonic spiking mode or bursting mode, which each activate a different gastric motor pattern [28]. Recent work has shown that the actions of AGR are influenced by other sensory neurons (Barriere et al., 2008) and that the effects of AGR firing depend on when it is active during gastric mill rhythms (Smarandache et al., 2008). 

Thus far, all of these physiological actions on gastric and pyloric network activity have been attributed to the long-loop polysynaptic reflex pathway through the anterior CoGs. No detailed study of the AGR morphology within the STG exists and, so far, projections in the STG neuropil have not been described. We now provide anatomical descriptions of AGR projections into the STG neuropil. 

## Methods

### Animals and dissections

Adult Jonah crabs (*Cancer borealis*) were obtained from Commercial Lobster (Boston, MA). All animals were kept in artificial seawater tanks at 10-13°C on a 12-hour light/12-hour dark cycle without food. Crabs were anesthetized on ice for 30 minutes. Dissections were performed as previously described [29] in chilled physiological saline consisting of: 440 mM NaCl, 11 mM KCl, 26 mM MgCl_2_, 13 mM CaCl_2_, 11 mM Trizma base, 5 mM maleic acid, pH 7.45. 

### Electrophysiology

The STNS was pinned down in a Sylgard-coated dish. The STG was desheathed and intracellular recordings from the somata and neuropil were made with 12-40 MΩ glass microelectrodes filled with 0.6M KSO_4_ and 20 mM KCl, amplified with 1x HS headstages and Axoclamp 2A and 2B amplifiers (Molecular Devices). Extracellular activity was recorded with stainless steel pin electrodes that were placed into petroleum jelly wells on the motor nerves and amplified and filtered with a differential amplifier (AM-systems). For identification of motor neurons, intracellular soma recordings were matched with extracellular recordings from the appropriate motor nerve. During the recording, the STNS was continuously superfused with chilled (9-13 °C) saline.

### Cell fills

Somata or axons were filled either with:

a 2% Lucifer Yellow CH dipotassium salt (LY; Sigma, catalog number L0144) in filtered water,
b 4% tetramethylrhodamine-dextran 3KDa, lysine fixable (TMR; Molecular Probes, catalog number D3308) in 0.2% KAc,
c 4% neurobiotin tracer (Vector Laboratories) in 50 mM Tris buffer and 0.5 M KCl, or
d 10 mM Alexa Fluor 568- or 594-hydrazide sodium salt in 200mM KCl (Molecular Probes, catalog numbers A10441 and A10442).

For all tracers, the tips of low-resistance glass electrodes (3-16 MΩ) were backfilled by capillary action for 10 minutes. For TMR, the back of the electrodes was filled with 2M KAc, leaving a small gap between the KAc and the TMR in the tip. Lucifer Yellow and Alexa Fluor-hydrazides were injected for 20-50 minutes with negative pulses of –3 to –11 nA of 0.5 s duration at 0.1-1 Hz until the fine neuropil processes of the cell were visible with a fluorescent microscope (Leica MF165 FC). TMR and Neurobiotin were injected for at least 50 minutes using 3 nA positive current pulses (neurobiotin) or 4-13 nA positive current pulses (TMR) of 0.5 s duration at 1 Hz. Unless otherwise stated, preparations were fixed with 3.5% paraformaldehyde in phosphate-buffered saline (PBS; 440 mM NaCl, 11 mM KCl, 10 mM Na_2_HPO_4_, 2 mM KH_2_PO_4_, ph 7.4) for 40 to 90 minutes at room temperature within 3 hours after filling with LY, neurobiotin and TMR and within 30 minutes after filling with Alexa Fluor-hydrazides. Preparations were washed with 0.01M PBS-T (0.1-0.3% Triton X-100 in PBS) and stored for 0–7 days at 4°C before processing. 

### Dye amplification

The LY signal was amplified by addition of a polyclonal rabbit anti-LY antibody (1:500; Molecular Probes) and the appropriate Alexa Fluor-conjugated secondary anti-rabbit antibody (1:500; Molecular Probes) during immunohistochemical processing. Neurobiotin was visualized by addition of streptavidin-conjugated Alexa Fluor dyes (1:500; Molecular Probes) to the secondary antibody mix (see next section).

### Immunohistochemistry

We used a rat monoclonal antibody against substance P antibody to label *Cancer borealis* tachykinin-related peptide (CabTRP; Accurate Chemical and Scientific, Westbury, NY, #NC1/34HL, 1:300 dilution) [30,31], a mouse monoclonal antibody against Drosophila synapsin GST-fusion protein (SYNORF1, [32]; Developmental Studies Hybridom Bank (Univ. Iowa), #5F10, 1:500 dilution), and a rabbit polyclonal antibody against Lucifer Yellow (Molecular Probes, catalog number A-5750, 1:1000 dilution). Antibody specificity for synapsin-like and CabTRP-like immunolabeling in *C. borealis* was previously demonstrated [30,31,33]. Antisera were diluted in 0.01M PBS-T at the appropriate concentration and applied overnight at room temperature, followed by 6x10 minutes washes with PBS-T.

For secondary detection, Alexa Fluor - conjugated goat polyclonal antibodies against mouse or rabbit IgG (H+L chains, highly cross-absorbed; Molecular Probes) were used to visualize immunoreactivity. The antibodies were used at concentration of 1:500 in PBS-T for 2–3 hours at room temperature. Preparations were then washed 4x15 minutes in PBS. STGs were then mounted on pre-cleaned slides (25x75x1mm, superfrost, VWR) in Vectashield (Vector Laboratories, Burlingame, CA), with 9mm diameter, 0.12mm depth silicone seal spacers (Electron Microscopy Sciences, Hatfield, PA) under #1.5 coverslips (Fisher Scientific).

### Statistics

AGR process lengths were analyzed in Excel (Microsoft). Significance was tested with t-tests in SigmaPlot. Errors are standard deviation. 

### Image acquisition and processing

Tiled stacks of confocal images were collected with a SP5 CLEM microscope using Leica Application Suite Advanced Fluorescence (LAS AF) software. For multiple labels, sequential imaging and narrow emission settings were used to prevent cross talk. Confocal images were acquired as tiles with a 63x glycerol objective (Leica HCX PL APO 63x / 1.3 GLYC CORR CS (21° C) at 2048x2048 or 1024x1024 resolution in 0.12 to 1.01 µm steps and subsequently aligned and stitched with a GUI-based MATLAB tool, written by Ted Brookings. Image stacks were converted to .ims files with Imaris 7.0–7.4 (Bitplane) filtered and down-sampled to a third of the resolution in the x and y dimensions. The Imaris Slice and Surpass modules were used for adjusting contrast and brightness and to display stacks as maximum-intensity projections or as blend mode projections. Subsets of the z-stacks were displayed in blend mode projections with reduced opacity to improve visualization of cell fills and protein distribution at the same time. Filament and volume reconstructions of cell fills were made with the Imaris Filament module in manual mode to determine branch lengths and branch volumes.

### Separation of Synapsin-like labeling within and around filled processes

Synapsin-like immunoreactive labeling within filled processes was isolated from the overall synapsin-like signal by using surface reconstructions of the filled cell as a mask. The surface reconstructions were performed with the Imaris Surface module using a manually set threshold for intensity to distinguish between background and cell fill. The overlap of the reconstructed cell surface mask and the synapsin-like immunoreactive signal was saved as a new signal channel (“synapsin-inside”). To capture synapsin-like labeling adjacent to the filled cell, this process was repeated with a new surface mask of the filled cell, this time generated by using a lower threshold of intensity, capturing the “glow” around the cell surface. This resulted in a mask that over-estimated the volume of the cell and allowed us to isolate synapsin-like labeling in the vicinity of the filled cell by subtracting the “synapsin-inside” signal from this new signal channel, using the “Channel Arithmetics” function in Imaris.

It is important to point out that a high sampling rate during image acquisition, ideally twice the resolution of the used objective (Nyquist rate), is critical to avoid loss during subsequent filtering and to allow sufficiently accurate surface reconstructions. 

## Results

### AGR projects into the synaptic neuropil

**Figure 1:**
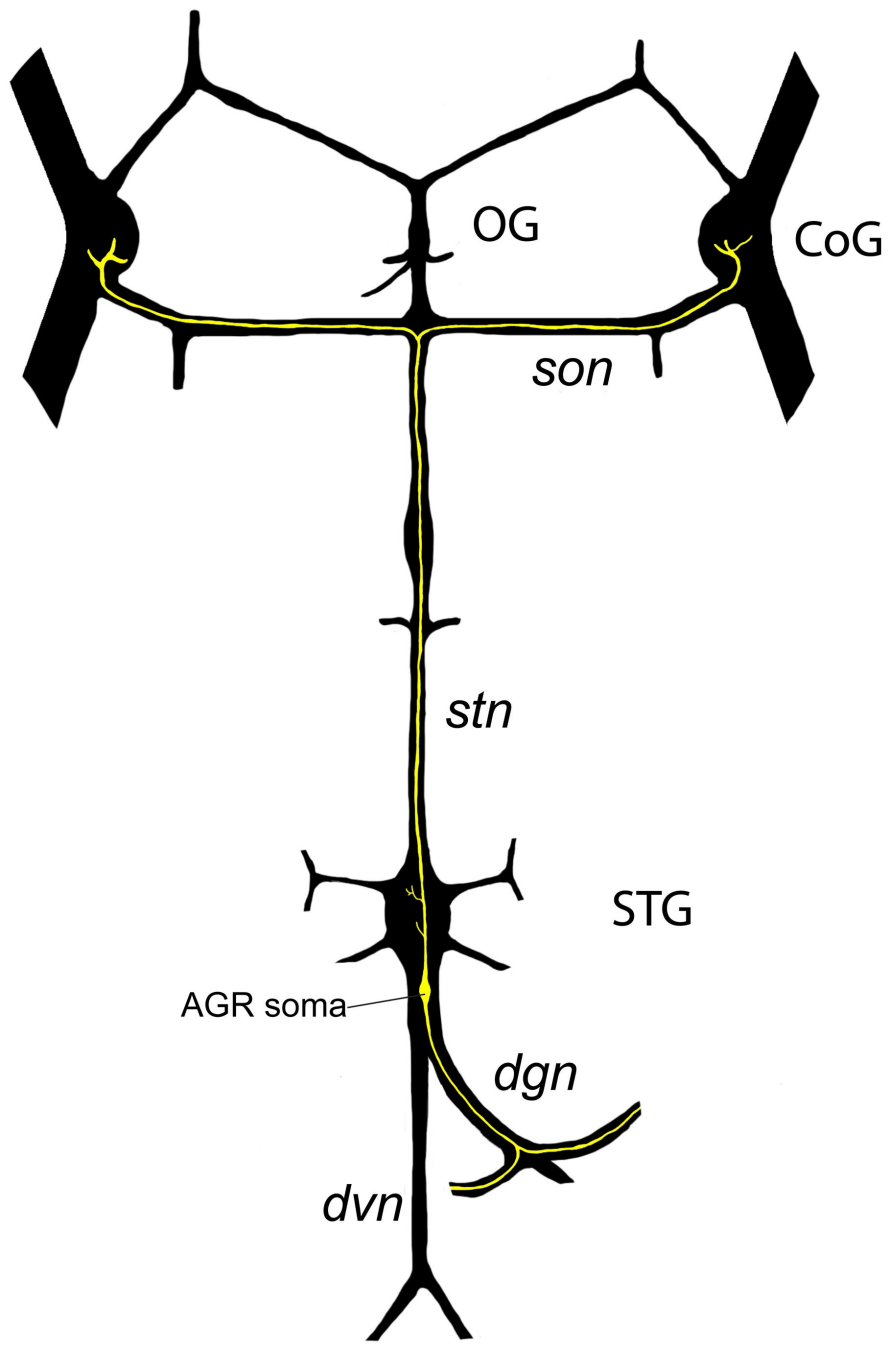
Schematic overview of the STNS, showing the location of the AGR neuron (yellow) in the *dvn*, its projections through the *dgn*, and its anterior projections through the *stn* into the commissural ganglia (CoG).

 We used dye fills to trace the AGR axon and its projections in the STG. The position of the midline bipolar AGR in the STNS has been previously described (Combes et al., 1995) (Figure 1). The position of the soma in different preparations is somewhat variable, as the soma can be found as much as 500 µm posterior to the STG in the dorsal ventricular nerve (dvn) or it can be seen ventrally below cell bodies in the posterior region of the STG, quite close to the STG neuropil. 

**Figure 2:**
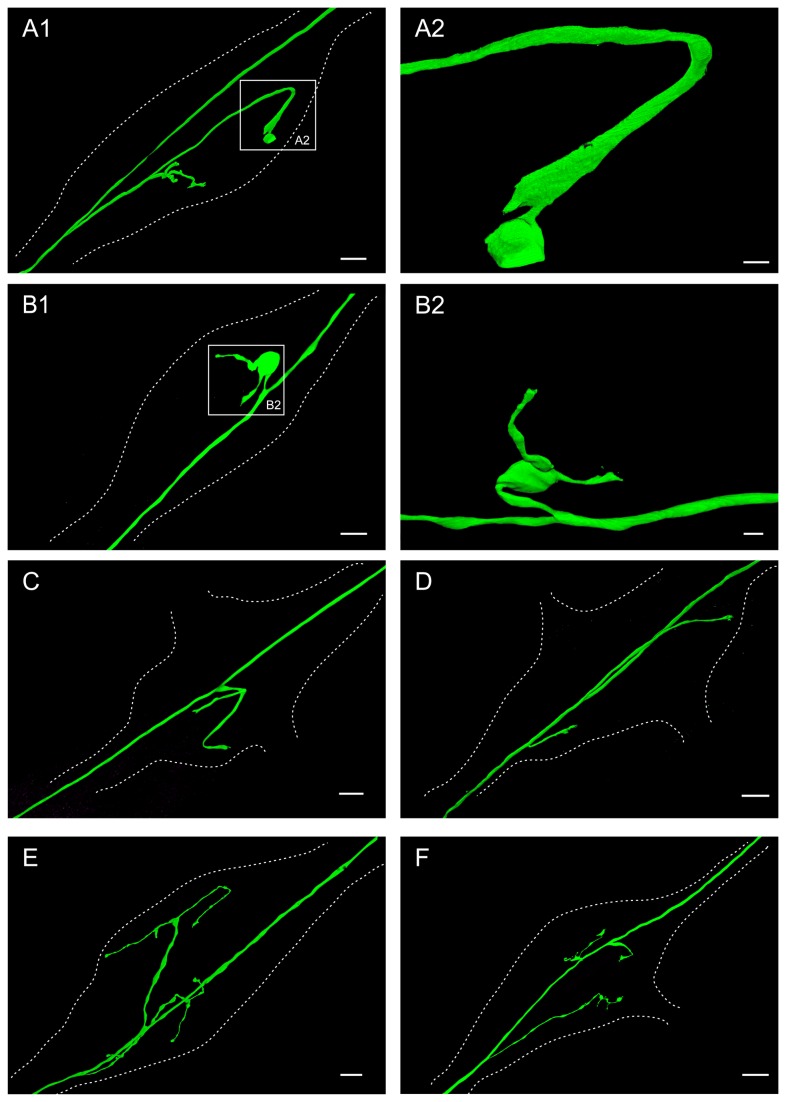
The AGR axon has a single (A-C), two (D-E) or three (F) projections from the main axon in the STG neuropil. Dotted lines outline the neuropil area of the ganglion in all parts of the figure. **A1**. Volume-rendered view of LY dye-filled AGR with single projection, branching further into four sub-branches in the STG neuropil. The AGR soma is located in the *stn* outside the lower-left corner of the frame. Blend mode projection of 8 merged confocal image stacks, each consisting of 84 optical slices (acquired at resolution of 0.067µm x 0.067µm x 0.378µm). Scale bar is 30µm. **A2** shows close-up of the boxed area in A1, showing the widened end of the AGR projection within the STG. Scale bar is 5µm. **B1**. Volume-rendered view of LY dye-filled AGR with single projection, branching further into two short sub-branches in the STG neuropil. The AGR soma is located in the *stn* outside the lower-left corner of the frame. Blend mode projection of 4 merged confocal image stacks, each consisting of 226 optical slices (acquired at resolution of 0.174µm x 0.174µm x 0.294µm). Scale bar is 30µm. **B2** shows a volume-rendered surface projection of the boxed area in B1 from a different angle, emphasizing the big balloon-like widening (not the soma) of the axonal AGR projection. Scale bar is 10µm. **C**. Volume-rendered view of LY dye-filled AGR with single projection, branching further into two sub-branches in the STG neuropil. The AGR soma is located in the *stn* outside the lower-left corner of the frame. Blend mode projection of 5 merged confocal image stacks, each consisting of 169 optical slices (acquired at resolution of 0.068µm x 0.068µm x 0.462µm). Scale bar is 30µm. **D**. Volume-rendered view of LY dye-filled AGR with two simple projections in the STG neuropil. The AGR soma is located in the *stn* outside the lower left corner of the frame. Blend mode projection of 18 merged confocal image stacks, each consisting of 115 optical slices (acquired at resolution of 0.183µm x 0.183µm x 0.504µm). Scale bar is 30µm. **E**. Volume-rendered view of LY dye-filled AGR with two projections, each branching further into two or three sub-branches in the STG neuropil. The AGR soma is located in the *stn* outside the lower-left corner of the frame. Blend mode projection of 10 merged confocal image stacks, each consisting of 264 optical slices (acquired at resolution of 0.179µm x 0.179µm x 0.38µm), ventral view of same preparation as Figure 3. Scale bar is 30µm. **F**. Volume-rendered view of LY dye-filled AGR with three projections, one of them branching further into two sub-branches in the neuropil. The AGR soma is located in the *stn* outside the lower-left corner of the frame. Blend mode projection of 9 merged confocal image stacks, each consisting of 229 optical slices (acquired at resolution of 0.168µm x 0.168µm x 0.252µm). Scale bar is 50µm.

We found AGR projections from the main axon into the neuropil of every STG that we examined (N =29) (Figure 2). Confocal imaging revealed AGR projections with varying length and branching pattern in the STG. The number of AGR projections into the STG neuropil varied from one to three and their morphology was remarkably diverse, ranging from minimally to complexly branched (Figure 2). Of the 29 dye-filled AGR neurons, 17 had one process branching off the main axon into the STG neuropil, 10 had two processes, and in two preparations, three projections into the STG were seen. Common features that were seen throughout many AGR cell fills were hook-shaped branches in the anterior part of the ganglion (Figure 2A) (N=13 out of 23 AGRs), branches that ended in claw-like processes (Figure 2B) (N=8 out of 23 AGRs), and bulbous swelling of AGR processes (Figure 2B) (N=18 out of 23 AGRs). 

We measured the branching characteristics in 17 AGRs that were imaged at high resolution (Table 1). In preparations with only a single AGR branch from the main axon, the process branched on average 3 times. Its length varied across preparations and averaged 240 ± 136 µm (standard deviation) (Figure 2A-C). The number of terminal segments was 1.7 ± 0.8 . AGR processes with two projections into the neuropil tended to be shorter on average (176 ± 95.4 µm for the first, p = 0.014, and 109 ± 94.41 µm for the second branch), with slightly more individual sub-branches (2.5 ± 1.3 terminal segments), but due to the large variance in branch numbers, this difference was not statistically significant (p = 0.160) (Figure 2D-F). There was also no significant conservation of total projection length when we normalized for neuropil length (average neuropil length = 544 ± 81.1 µm, N = 17) and compared the summed branch length in preparations with one or two processes (p = 0.69) (Table 1). However, in preparations with two projections into the neuropil, as shown in Figure 2 D and E, the more posterior (closer to the AGR soma) process was significantly shorter than the anterior projection (89 ± 73 µm versus 168 ± 99 µm, respectively, p=0.003).

**Table 1:** AGR branching characteristics.

Number of branches	N	1^st^ branch length	1^st^ branch length, normalized for neuropil size	2^nd^ branch length	2^nd^ branch length, normalized for neuropil size	3^rd^ branch length	3^rd^ branch length, normalized for neuropil size	Total branch length	Total branch length, normalized for neuropil size
1	8	240±136.2	235±118.3	—	—	—	—	241±136.2	235±118.3
2	7	109±94.1	89±73.3	176±95.4	168±98.6	—	—	286±94.8	258±85.9
3	2	175±219.2	169±202.3	72±31.8	77±18.1	60±56.6	60±48.6	307±103	306±89.7

**Figure 3:**
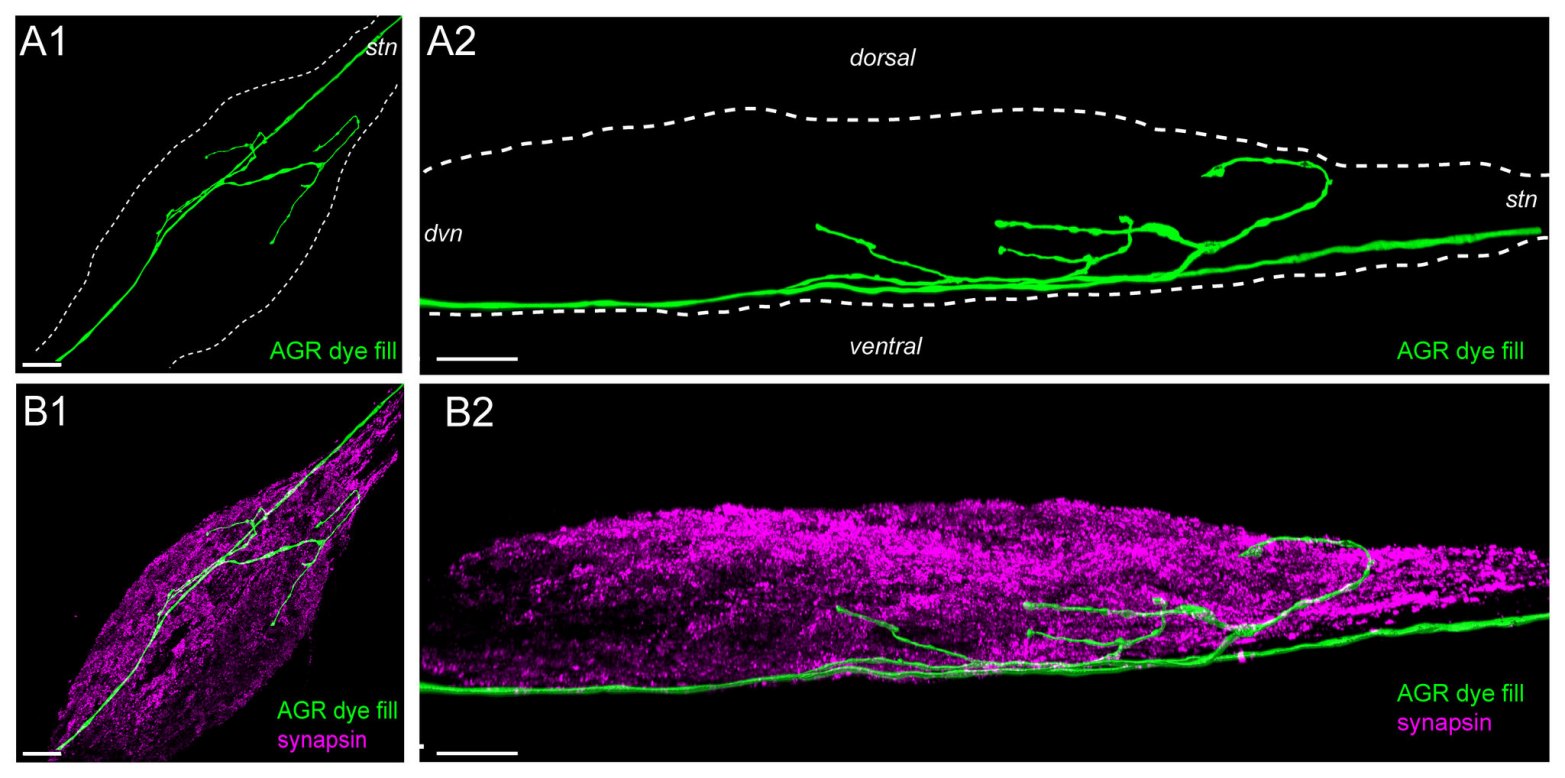
The AGR axon typically runs along the ventral surface of the STG and projects dorsally into the neuropil. **A1**. Volume-rendered dorsal view of LY dye-filled AGR (green), projected along the dorso-ventral axis. **A2**. Lateral, maximum intensity projection of the same ganglion. **B1**. Double labeling with anti-synapsin antibody (purple) reveals the synaptic neuropil in the STG. **B2**. Lateral projection of the anti-synapsin labeled ganglion shows the ventrally located AGR axon and its dorsal projections into the synaptic neuropil. Blend mode (A1, B1 and B2) and maximum intensity (A2) projections of 10 merged confocal image stacks, each consisting of 264 optical slices (acquired at resolution of 0.179µm x 0.179µm x 0.38µm). Scale bar is 50µm.

Another striking anatomical feature was the localization of the AGR branches with regard to the ganglion architecture. The STG somata are located in a cluster around the neuropil in the posterior region of the ganglion. These neurons send a primary neurite towards the center of the ganglion (coarse neuropil), where it branches into incrementally smaller processes and one or more axons that leave the ganglion through the connected nerves. The smallest processes of the STG neurons are located in-between the center of the ganglion and the cell bodies on the outside, where they form the fine or synaptic neuropil, a shell-like structure around the coarse neuropil that is the interior of the ganglion (Figure 3) [34]. The fine neuropil is the area where synaptic interactions take place [35]. It can be labeled with antibodies against presynaptic proteins such as synapsin (synorf) [14,32,33,36]. We found that the AGR axon was sometimes off-center to the right or left but always ran on the most ventral side of the STG (n=27) (Figure 3AB). From there, the AGR projected dorsally through the coarse neuropil at the center of the STG and into the dorsal fine neuropil (Figure 3B). 

### Localization of candidate contact sites between AGR other cells

We wanted to know if the AGR projections came into close contact with other STG neurons. For this we performed double fills of AGR and other STG cells, focusing on neurons that are part of the gastric mill network, where AGR stimulation has the strongest effects on network activity *in vivo* and *in vitro* [24,25,37,38].

**Figure 4:**
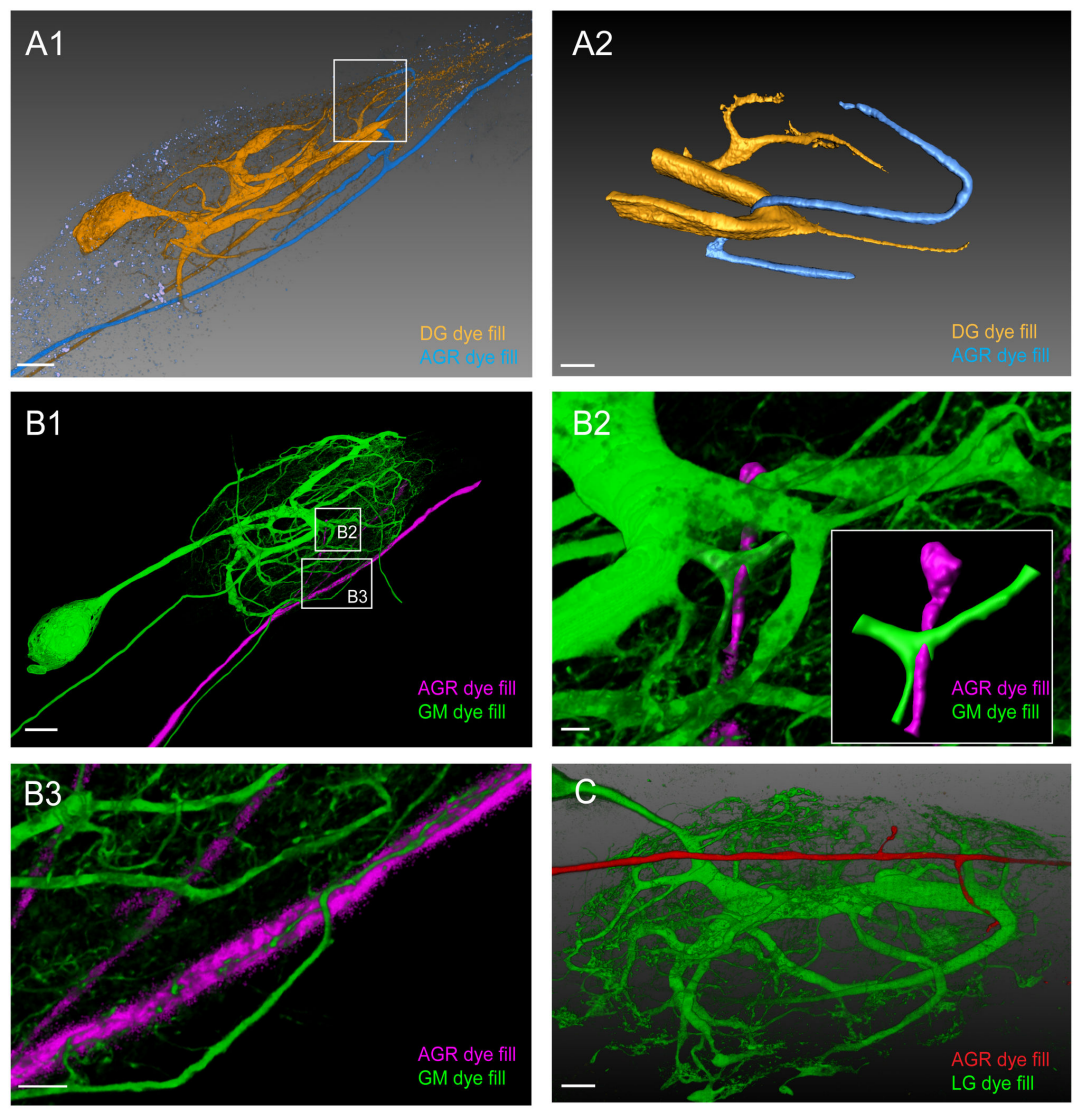
Double fills of the AGR and other STG neurons identify potential synaptic partners. **A1**. Double fill of the DG neuron with TMR (yellow) and the AGR neuron with LY (blue) shows candidate contact sites in the neuropil. Lateral blend mode projection of 18 merged confocal image stacks, each consisting of 133 optical slices (acquired at resolution of 0.177µm x 0.177µm x 1.007µm). Scale bar is 40µm. **A2**. Reconstructed surface visualization of the DG-AGR candidate contact site in the coarse neuropil, reconstructed from the same data set as A1. Scale bar is 10µm. **B1**. Double fill of a GM neuron with neurobiotin (green) and the AGR neuron with alexa Fluor 594-hydrazide (magenta). Blend mode projection of 18 merged confocal image stacks, each consisting of 185 optical slices (acquired at resolution of 0.088µm x 0.088µm x 0.504µm), background-subtracted. Scale bar is 50µm. **B2**. Close-up of candidate contact site in the coarse neuropil. Scale bar is 5µm. A reconstructed surface visualization of the contact site (inset in B2) allows a clearer view. **B3**. Close-up of candidate contact site between fine neuropil processes of GM and the AGR axon. Scale bar is 10µm. **C**. Double fill of the LG neuron with LY (green) and the AGR neuron with alexa Fluor 594-hydrazide (red) shows apparent contact site between the LG primary neurite and an AGR process. Blend mode projection of 8 merged confocal image stacks, each consisting of 155 optical slices (acquired at resolution of 0.177µm x 0.177µm x 0.336µm), background-subtracted. Scale bar is 30µm.

Double fills of the DG neuron and AGR in three preparations revealed several areas of apparent close contact at the level of resolution available with the confocal microscope (Figure 4A). We performed surface reconstructions of one of these pairs to gain a better view of the apparent contact sites. The most striking candidate contact point was not in the synaptic (fine) neuropil, but in the coarse neuropil in the center of the ganglion, where a large diameter process of DG was seen seemingly wrapped around an AGR projection. In addition, another candidate contact site of AGR with a very fine DG process was seen in the synaptic neuropil (Figure 4A). Other cells that came into close contact with AGR whenever we performed double fills were at least one of the four gastric mill neurons (GM) (n=3) and the single lateral gastric neuron (LG) (n=5). Closer inspection of a double fill of the GM neuron and AGR in two preparations showed one single candidate site of apparent contact between two processes (Figure 4B), clearly seen in a surface reconstruction of the contact site in the insert in Figure 4B2. In the same preparation, very fine processes (~1µm diameter) of the GM neuron also wrapped around the AGR axon (Figure 4B3). Figure 4C shows a double fill of AGR and LG with candidate contact sites on the larger processes in the coarse neuropil. Similar candidate contact sites were seen in the neuropil of five ganglia with LG and AGR double fills. 

### Descending projection fibers

**Figure 5:**
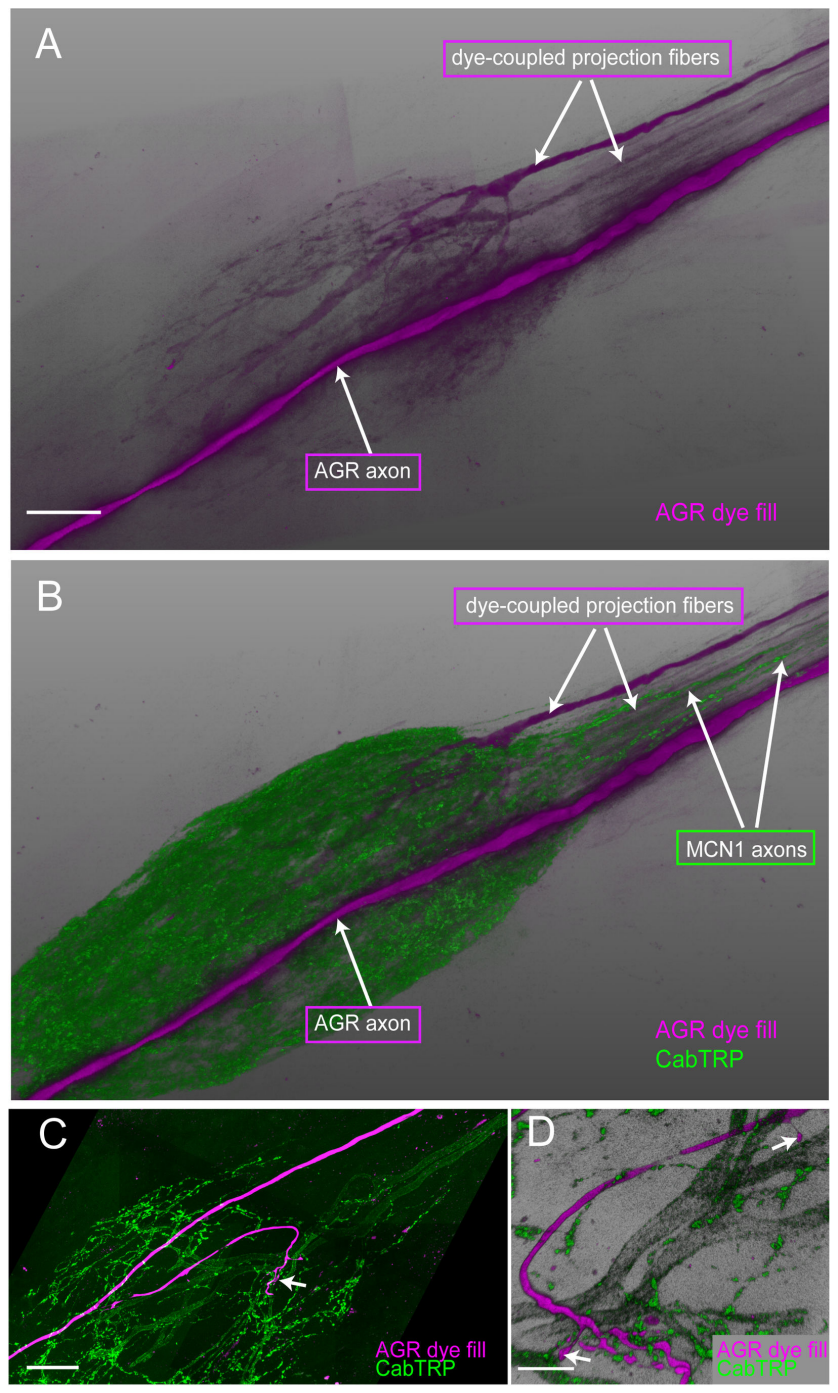
Dye coupling reveals putative electrically coupled neurons. **A**. Long-term (2 hours) LY dye-fill of the AGR neuron (magenta) revealed dye-coupled descending STN fibers. **B**. Double labeling for CabTRP in the same preparation shows that the dye-coupled STN fibers were not MCN1 projections. A and B are blend mode projections of 12 merged confocal image stacks, each consisting of 200 optical slices (acquired at resolution of 0.187µm x 0.187µm x 0.504µm). Scale bar is 50µm. **C**. AGR processes (LY dye-filled, magenta) are in close apposition to projections from the MCN1 neurons that were labeled with an anti-substance P antibody (green). Blend mode projection of 12 merged confocal image stacks, showing a 47µm thick mid-section of the ganglion (127 of 210 optical slices, acquired at resolution of 0.187µm x 0.187µm x 0.713µm). Scale bar is 50µm. **D**. A close-up of the neuropil shows a claw-like ending of the LY dye-filled AGR projection (magenta) in close contact with fine MCN1 processes (green), and two distinct AGR terminals in apparent contact with larger-diameter MCN1 processes (arrows). Blend mode projection of 24µm thick mid-section in the same preparation as 4B, rotated 180° around the dorso-ventral axis (66 of 210 optical slices, acquired at resolution of 0.187µm x 0.187µm x 0.713µm). Scale bar is 15µm.

Approximately 25 pairs of descending neurons project from the CoGs and the OG through the stomatogastric nerve *stn* to the STG [39], where modulator release from their terminals shapes the activity of gastric and pyloric neurons [40-43]. In four preparations, when we filled AGR with Lucifer Yellow for an extended period of time (2-3 hours), we found weak dye spread from AGR to processes that were similar in shape to described projection neurons (Figure 5A). Interestingly, when we repeated the experiment with neurobiotin, a molecule that passes through gap junctions in the crustacean nervous system, we did not see any coupling to other cells. We wanted to know if the dye-coupled processes in the STG were projections of the Modulatory Commissural Neuron 1 (MCN1), a pair of modulatory cells in the CoG that are activated and regulated by AGR activity via the long-loop pathways through the *stn* and CoGs, and elicit a gastric mill rhythm [37,42-44]. MCN1 contains three modulatory peptides in its projections in the STG; one of them is a tachykinin-like peptide, CabTRP1a. As MCN1 is the only source of tachykinin-like peptide in the STG, the two MCN1 projections in the STG can be specifically labeled with an anti-substance P antibody, which recognizes tachykinin-like peptides across many different species [30,31]. We performed immunolabeling of CabTRP to visualize MCN1 terminals in three ganglia with dye-spread from AGR to *stn* processes. In one of these preparations, a dye-coupled *stn* process labeled positive for CabTRP and therefore was likely MCN1. In the other two preparations, the two MCN1 projections did not coincide with the dye-coupled process in the STN (Figure 5B). However, when we performed double labeling of AGR and CabTRP in ganglia without dye-spread, we consistently found at least one of the AGR branches in close vicinity or wrapped around an MCN1 axon before it arborized in the neuropil (Figure 5C-D) (N=7 out of 7). 

**Figure 6:**
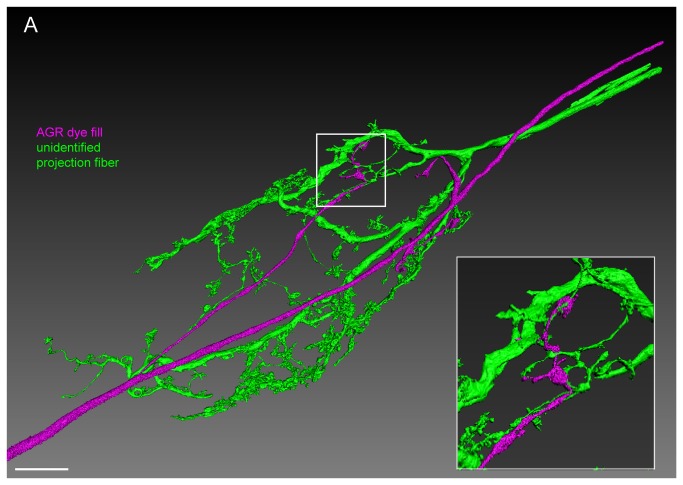
The AGR projections are in close apposition with projections from descending fibers in the STG neuropil. Double fill of an unidentified STN process with LY (green) and the AGR neuron with alexa Fluor 594-hydrazide (magenta) shows close apposition of processes over a large area in the STG neuropil. Reconstructed surface visualization of 9 merged confocal image stacks, each consisting of 229 optical slices (acquired at resolution of 0.168µm x 0.168µm x 0.252µm). Scale bar is 50µm. Insert shows close-up of boxed area, revealing apparent contact sites between the AGR projection terminals and fine processes of the unidentified projection neuron.

Lastly, we performed double recordings of AGR and unidentified processes in the stomatogastric nerve (stn) (Figure 6). We recorded from 1-5 *stn* axons per ganglion. In the majority of ganglia when both cells were monitored intracellularly, we found no *stn* processes that were electrically coupled to AGR (N=7). However, on one occasion electrical coupling upon current injection into either AGR or the unidentified STN process was seen and the *stn* process was dye-filled. The insert in Figure 6A shows a surface reconstruction of an area of close contact between AGR and this descending unidentified *stn* process in the fine neuropil. Unfortunately, we were not able to subsequently find, record from, or fill the same *stn* projection in other ganglia. 

### Putative chemical synapses of AGR in the STG

**Figure 7:**
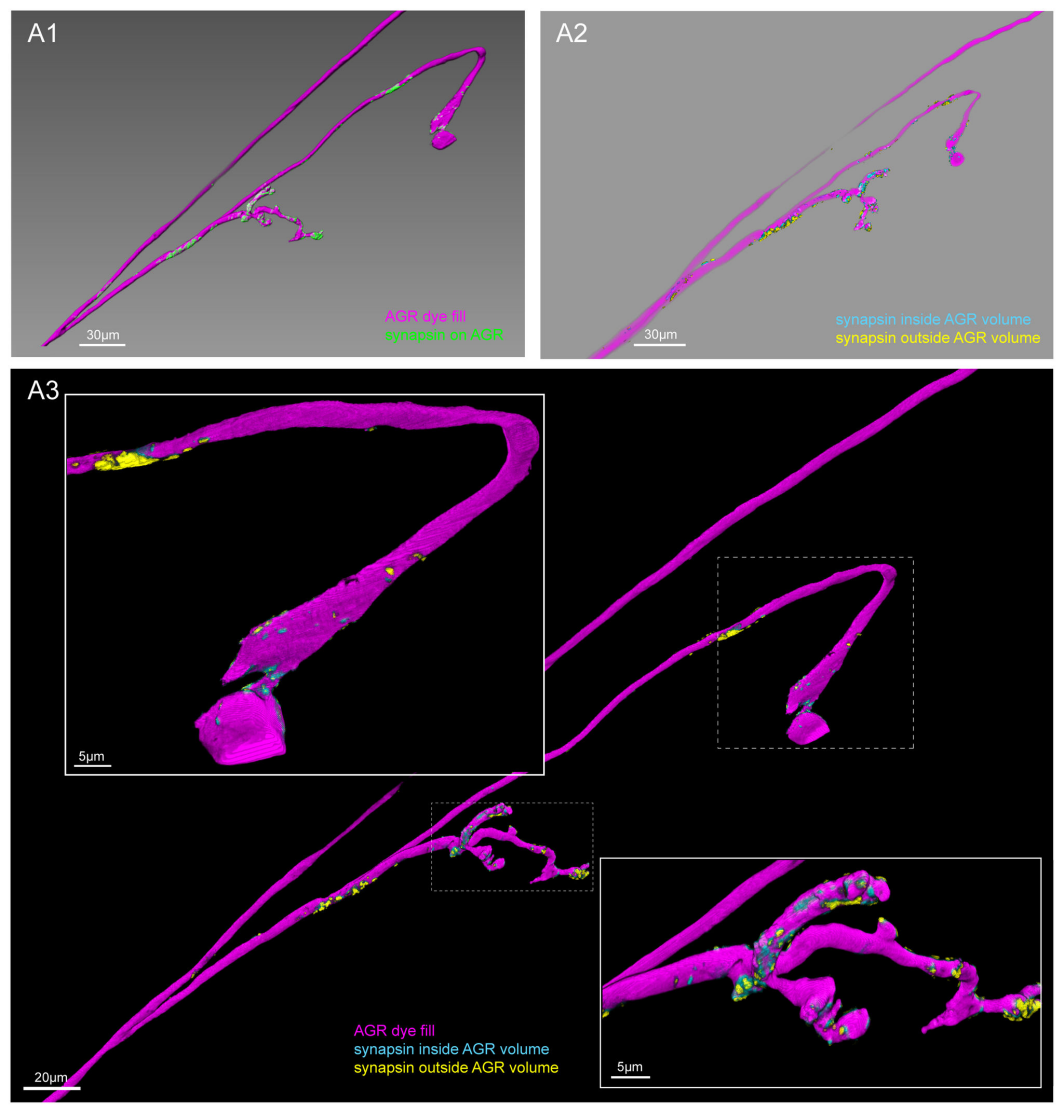
Clustered sites of putative chemical synapses are found in the AGR projections, but typically not in the AGR axon. **A1**. Double labeling with an antibody against synapsin reveals patches of immuno-labeling on the LY dye-filled AGR projections. The image was processed to only show synapsin labeling in the AGR (see methods). Scale bar is 30µm. **A2**. Putative pre- and postsynaptic sites in the AGR projections in the same preparation. Different masking methods allow distinguishing between potentially presynaptic and postsynaptic sites in the AGR neuron (see methods). Synapsin labeling that mostly overlapped with the volume of the reconstructed AGR surface was classified as putative pre-synaptic (blue), and is found predominantly in the distal parts of the AGR process. Synapsin labeling that mostly overlapped with a thin shell around the AGR neuron was interpreted to be located in the processes of adjacent cells, marking putative post-synaptic sites in the AGR neuron (yellow). Scale bar is 30µm. **A3**. Overlay of the putative pre-and postsynaptic sites with the AGR projection (magenta). The close-up in the inserts reveals the distinct clustering in putative pre- and postsynaptic sites of the AGR projection and axon. Putative pre-synaptic sites in the AGR neuron are blue and putative post-synaptic sites are yellow. A1-A3 are blend mode projections of the same data set of 8 merged confocal image stacks, each consisting of 84 optical slices (acquired at resolution of 0.067µm x 0.067µm x 0.378µm).

We next carefully examined the distribution of potential synapses in AGR cell fills. We used surface reconstructions to separate synapsin-like immunoreactivity in the AGR from the signal in the rest of the synaptic neuropil (see methods section). Figure 7A shows the isolated synapsin signal overlayed with the AGR cell fill. The AGR axon itself was almost entirely devoid of synapsin-like immunoreactivity, while the surface of the AGR branches in the STG neuropil showed clustered patches of synapsin-like labeling, mostly in the more distal part of the projections (Figure 7A). In preparations with multiple sub-branches, the synapsin-like signal was typically more concentrated in a subset of 1-2 branches. 

We wanted to determine if the patches of synapsin-like labeling were likely pre-or postsynaptic sites in the AGR neuron. Typically, confocal microscopy cannot clearly separate these two. However, the method we used - generating surface reconstructions and using them as masks to filter the synapsin signal - allowed us to separate the signal into putative pre-and postsynaptic sites. Our masking method picked up any voxel of synapsin-like signal that overlapped with the cell fill. However, the margins of a cell are somewhat ambiguous with traditional confocal microscopy methods and depend on the gain and chosen thresholds for signal cut-off. We used this to our advantage by creating a second surface reconstruction of the AGR fill, using lower thresholds and thus generating a volume that overestimated the boundaries of the cell to the best of our estimates. We classified synapsin-like immunoreactivity that was found within only the first AGR reconstruction as putative pre-synaptic (blue in Figure 7A2 and A3), while patches of synapsin-like labeling in the second, wider volume reconstruction of AGR were interpreted to be putatively post-synaptic (yellow in Figures 7B and C). This method revealed a non-uniform distribution with distinct clusters of primarily presynaptic or postsynaptic sites in the AGR fill. Putative pre-synaptic sites were predominantly found in the distal parts of the AGR projections. Putative post-synaptic sites were fewer and found closer to the axon branch point or in the mid-section of the projections (Figure 7A3). The bulging swellings that were often seen showed little synapsin labeling (Figure 7A3) (N=5 out of 7). 

**Figure 8:**
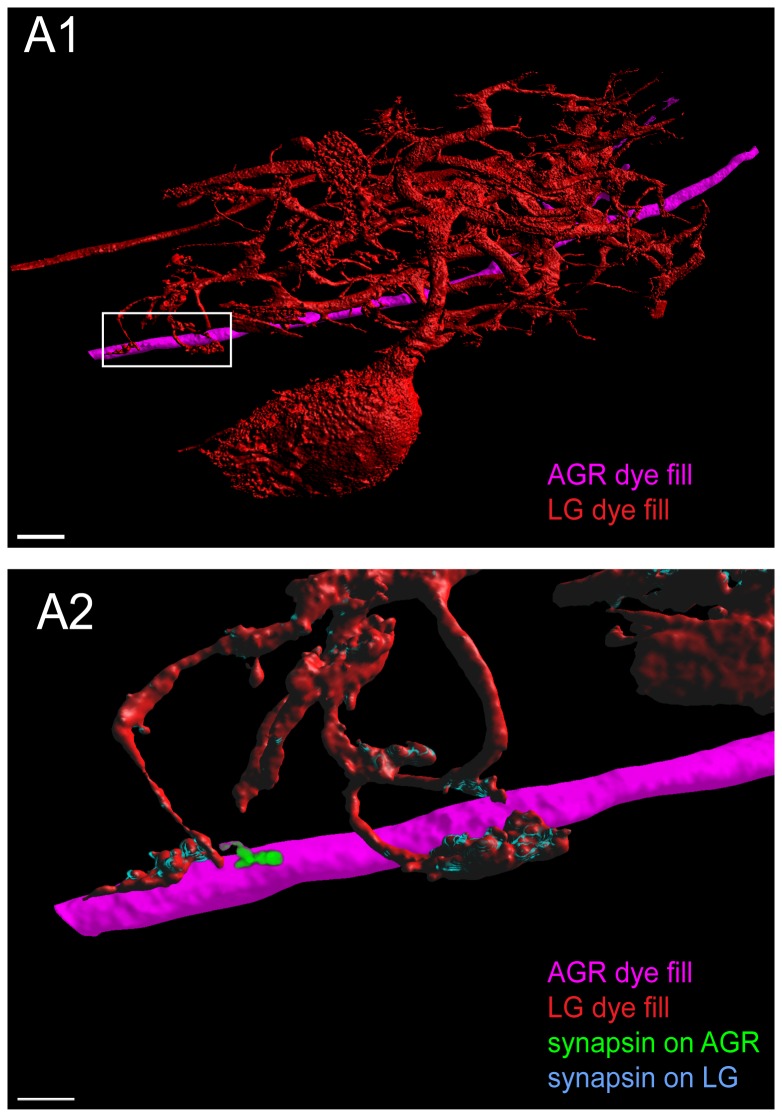
Putative chemical synapses at a contact site between LG and AGR. **A1**. Double fill of the LG neuron with LY (red) and the AGR neuron with alexa Fluor 594-hydrazide (magenta) shows apparent contact sites of LG processes wrapping around very short stubby projections on the AGR axon. **A2**. Higher magnification of the box in A1 shows adjacent patches of synapsin immunoreactivity in the LG and in the AGR neuron at their contact site. The image was processed to show synapsin labeling in the LG neuron in blue and synapsin labeling in the AGR neuron in green. A1 and A2 are reconstructed surface visualizations of the same data set of 12 merged confocal image stacks, each consisting of 182 optical slices (acquired at resolution of 0.158µm x 0.158µm x 0.504µm).

We performed triple-labeling in three preparations in which we dye-filled AGR with one dye and either the LG, DG or GM neuron with a second dye before we performed immunohistochemistry for anti-synapsin labeling. Figure 8A1 shows an example of a candidate contact site between an LG neuron and a very short process on the AGR axon. A careful examination showed adjacent patches of synapsin immunoreactivity at this site, one located in the LG terminal and one in the adjacent AGR neuron at their contact site (Figure 8A2). Similarly, we found synapsin-like labeling at one candidate contact site between AGR and GM in the fine neuropil but none at the DG-AGR candidate contact sites in the coarse neuropil (not shown). 

## Discussion

For decades, the circuits of the crustacean STNS have served as a model system for understanding small network behavior and organization. All of the motor neurons and interneurons in the STG and their synaptic connections have been identified. Neuromodulation, as well as sensory feedback, to the circuits have been extensively studied [6,17,45,46]. Therefore, it was surprising to discover projections of a well described sensory neuron within the STG that had gone unobserved until now [19,21-24,26,37]. Aside from the potential functional implications of additional AGR connections in the STNS circuits, these relatively simple projections provide an excellent opportunity to examine aspects of neuronal morphology.

### Variability of morphological features in identified neurons across animals

The AGR neuron uniformly sent projections into the STG, which then showed remarkable diversity in branching length, order, and shape. However, some morphological features of this neuron were consistent, like the stereotyped axonal projection along the ventral midline of the STG and the branch projections into the dorsally located synaptic neuropil. It seems reasonable to infer that stereotyped features of the AGR, as it is the case for any neuron, are either dictated by developmental factors or reflect a functionally important aspect [9,12,47]. The midline position of the AGR axon is most likely a consequence of developmental constraints; the stereotyped projection into the synaptic neuropil indicates a functional implication and suggests that the AGR branches could be sites of synaptic interaction and might in fact be part of a reflex pathway. The variance in branching structure is not necessarily surprising, considering that the position of individual STG neuron somata varies from animal to animal [14,15]). 

Consequently, the distance between the AGR axon and potential synaptic partners would vary to a similar degree and require shorter or longer branches across animals. However, considering that there are AGR neurons with only two simple projections and others with four levels of branching order, one cannot but wonder how differently signals are integrated in cells with varying number of processes. Alternatively, neuron morphology may be yet another complex case where multiple solutions can be found that lead to the same network output [48]. This would not be the first time to show that variability exists in the morphology of single cell types [14,49]. 

### AGR projections as candidate sites of synaptic contact

Obviously, the methods employed here can never unambiguously demonstrate the presence of synaptic connections; higher resolution electron microscope studies, the gold standard for synaptic localization, would be needed to do so. That said, the methods used here provide invaluable clues to where synapses are likely to be, and, just as importantly, where they are not likely to be. Given the large size of the STG neurons and neuropil and the relative paucity of candidate contact sites, it is clear from this work that determining the extent to which these candidate contact sites are true synapses will require correlated light and electron microscope studies.

The candidate contact sites between AGR and the STG terminals of MCN1, as well as the dye coupling between AGR and an unidentified descending neuron, suggest that AGR may make direct electrical and/or chemical synapses with the terminals of descending modulatory neurons. If so, AGR activity might change the modulatory state of the STG circuits through these connections. A previous study showed that presynaptic inhibition of the MCN1 terminals in the STG by another muscle receptor, the gastropyloric receptor (GPR), can contribute to slowing the gastric rhythm [5]. It is interesting that the number of individual candidate contact sites between AGR and putative synaptic partners was generally very low, ranging from one to three. In agreement with this, it has been suggested that only few sites of synaptic contact exist between two identified motor neurons of the pyloric network in the European lobster [50]. This pattern of sparse synaptic contact sites supports the idea that dendritic integration in STG neurons is not evenly distributed across the neuropil but rather compartmentalized, as suggested previously in this system by analysis of motor neuron morphology and sub-cellular receptor distribution [15,16,51]. 

It is well understood that conventional confocal images lack the resolution needed to characterize perfectly the boundaries of a filled process. We exploited this limitation to develop a masking image analysis method that enhances our ability to determine whether a label is likely presynaptic or postsynaptic. While we do not argue that this method is foolproof, its use segregates stained structures in a manner unlikely to randomly occur. For example, this method indicates that the distribution of putative pre- and postsynaptic sites appeared to be clustered in different preferred locations along the AGR neuron. 

Putative presynaptic sites were virtually always found in the distal parts of AGR branches. While these could be sites where AGR directly synapses with STG neurons, electrophysiological experiments of removing descending input from anterior ganglia that are well known to act on AGR, have shown little evidence for direct synapses. Though depolarizing AGR in an intact preparation evokes activity in gastric neurons, current injection into the AGR after cutting the *stn* did not change the activity or evoke postsynaptic potentials in any gastric or pyloric STG neuron. Alternatively, AGR could presynaptically inhibit or facilitate synapses within the STG. Interestingly, presynaptic inhibition of AGR itself appears to be one of the mechanisms that integrates sensory input from another sensory receptor, the PSR neurons, at the CoG synapses of the European lobster [20]. We found that putative postsynaptic sites in the same processes were preferentially located between the axonal branch point and putative presynaptic sites. Given the small diameter of the AGR branches, it is conceivable that synaptic transmission at the AGR terminals might be regulated through input at these putative postsynaptic sites. To influence AGR firing itself however, input at these sites would require second messenger activation because of their remote location from any of the AGR spike initiation zones [21,25]. 

The fact that the AGR projections in the STG have gone uncharacterized for so long in a system whose electrophysiological properties have been extensively studied, emphasizes the importance of detailed morphological studies in combination with functional examination. 

## References

[1] Pearson KG (1995) Proprioceptive regulation of locomotion. Curr Opin Neurobiol 5: 786–791. doi:10.1016/0959-4388(95)80107-3. PubMed: 8805415. 8805415

[2] Clarac F, Cattaert D, Le Ray D (2000) Central control components of a “simple” stretch reflex. Trends Neurosci 23: 199–208. doi:10.1016/S0166-2236(99)01535-0. PubMed: 10782125. 10782125

[3] Bässler U, Buschges A (1998) Pattern generation for stick insect walking movements--multisensory control of a locomotor program. Brain Res Brain. Res Rev 27: 65–88. doi:10.1016/S0165-0173(98)00006-X. 9639677

[4] Büschges A (2012) Lessons for circuit function from large insects: towards understanding the neural basis of motor flexibility. Curr Opin Neurobiol, 22: 1–7. doi:10.1016/j.conb.2012.02.003. PubMed: 22386530. 22386530

[5] Beenhakker MP, DeLong ND, Saideman SR, Nadim F, Nusbaum MP (2005) Proprioceptor regulation of motor circuit activity by presynaptic inhibition of a modulatory projection neuron. J Neurosci 25: 8794–8806. doi:10.1523/JNEUROSCI.2663-05.2005. PubMed: 16177049. 16177049 PMC6510986

[6] Blitz DM, Nusbaum MP (2011) Neural circuit flexibility in a small sensorimotor system. Curr Opin Neurobiol, 21: 1–9. doi:10.1016/j.conb.2011.05.019. PubMed: 21208796. 21689926 PMC3177960

[7] Windhorst U (2007) Muscle proprioceptive feedback and spinal networks. Brain. Research Bulletin 73: 155–202. doi:10.1016/j.brainresbull.2007.03.010. 17562384

[8] Clarac F, Cattaert D (1999) Functional multimodality of axonal tree in invertebrate neurons. J Physiol Paris 93: 319–327. doi:10.1016/S0928-4257(00)80060-1. PubMed: 10574121. 10574121

[9] Goodman CS (1996) Mechanisms and molecules that control growth cone guidance. Annu Rev Neurosci 19: 341–377. doi:10.1146/annurev.ne.19.030196.002013. PubMed: 8833447. 8833447

[10] Wu Z, Sweeney LB, Ayoob JC, Chak K, Andreone BJ et al. (2011) A Combinatorial Semaphorin Code Instructs the Initial Steps of Sensory Circuit Assembly in the Drosophila CNS. Neuron 70: 281–298. doi:10.1016/j.neuron.2011.02.050. PubMed: 21521614. 21521614 PMC3095019

[11] Prasad T, Weiner JA (2011) Direct and Indirect Regulation of Spinal Cord Ia Afferent Terminal Formation by the γ-Protocadherins. Front - Journal of Mol Neuroscience 4: 1–12. doi:10.3389/fnmol.2011.00054. PubMed: 21441980. PMC3250626 22275881

[12] Jessell TM, Sürmeli G, Kelly JS (2011) Motor Neurons and the Sense of Place. Neuron 72: 419–424. doi:10.1016/j.neuron.2011.10.021. PubMed: 22078502. 22078502

[13] Katsuki T, Ailani D, Hiramoto M, Hiromi Y (2009) Intra-axonal patterning: intrinsic compartmentalization of the axonal membrane in Drosophila neurons. Neuron 64: 188–199. doi:10.1016/j.neuron.2009.08.019. PubMed: 19874787. 19874787

[14] Bucher D, Johnson CD, Marder EE (2007) Neuronal morphology and neuropil structure in the stomatogastric ganglion of the lobster, Homarus americanus. J Comp Neurol 501: 185–205. doi:10.1002/cne.21169. PubMed: 17226763. 17226763

[15] Wilensky AE, Baldwin DH, Christie AE, Graubard K (2003) Stereotyped neuropil branching of an identified stomatogastric motor neuron. J Comp Neurol 466: 554–563. doi:10.1002/cne.10903. PubMed: 14566949. 14566949

[16] Oginsky MF, Rodgers EW, Clark MC, Simmons R, Krenz W-DC et al. (2010) D(2) receptors receive paracrine neurotransmission and are consistently targeted to a subset of synaptic structures in an identified neuron of the crustacean stomatogastric nervous system. J Comp Neurol 518: 255–276. doi:10.1002/cne.22225. PubMed: 19941347. 19941347 PMC3956453

[17] Stein W (2009) Modulation of stomatogastric rhythms. J Comp Physiol A Neuroethol Sens Neural Behav Physiol, 195: 989–1009. doi:10.1007/s00359-009-0483-y. PubMed: 19823843. 19823843

[18] Larimer JL, Kennedy D (1966) Visceral afferent signals in the crayfish stomatogastric ganglion. J Exp Biol 44: 345–354. PubMed: 5957028. 5957028 10.1242/jeb.44.2.345

[19] Combes D, Simmers J, Nonnotte L, Moulins M (1993) Tetrodotoxin-sensitive dendritic spiking and control of axonal firing in a lobster mechanoreceptor neurone. J Physiol (Lond) 460: 581–602. 8487209 10.1113/jphysiol.1993.sp019488 PMC1175230

[20] Barrière G, Simmers J, Combes D (2008) Multiple mechanisms for integrating proprioceptive inputs that converge on the same motor pattern-generating network. J Neurosci 28: 8810–8820. doi:10.1523/JNEUROSCI.2095-08.2008. PubMed: 18753383. 18753383 PMC6670814

[21] Combes D, Simmers J, Moulins M (1995) Structural and functional characterization of a muscle tendon proprioceptor in lobster. J Comp Neurol 363: 221–234. doi:10.1002/cne.903630205. PubMed: 8642071. 8642071

[22] Smarandache CR, Stein W (2007) Sensory-induced modification of two motor patterns in the crab, Cancer pagurus. J Exp Biol 210: 2912–2922. doi:10.1242/jeb.006874. PubMed: 17690240. 17690240

[23] Hedrich UB, Stein W (2008) Characterization of a descending pathway: activation and effects on motor patterns in the brachyuran crustacean stomatogastric nervous system. J Exp Biol 211: 2624–2637. doi:10.1242/jeb.019711. PubMed: 18689416. 18689416

[24] Smarandache CR, Daur N, Hedrich UBS, Stein W (2008) Regulation of motor pattern frequency by reversals in proprioceptive feedback. Eur J Neurosci 28: 460–474. doi:10.1111/j.1460-9568.2008.06357.x. PubMed: 18702718. 18702718

[25] Daur N, Nadim F, Stein W (2009) Regulation of motor patterns by the central spike-initiation zone of a sensory neuron. Eur J Neurosci 30: 808–822. doi:10.1111/j.1460-9568.2009.06866.x. PubMed: 19686469. 19686469 PMC2885921

[26] Simmers J, Moulins M (1988) A disynaptic sensorimotor pathway in the lobster stomatogastric system. J Neurophysiol 59: 740–756. PubMed: 3367197. 3367197 10.1152/jn.1988.59.3.740

[27] Combes D, Simmers J, Moulins M (1997) Conditional dendritic oscillators in a lobster mechanoreceptor neurone. J Physiol (Lond) 499 ( 1): 161–177. 9061647 10.1113/jphysiol.1997.sp021918 PMC1159344

[28] Combes D, Simmers J, Meyrand P, Moulins M (1995) Motor programme switching by a lobster mechanoreceptor neurone in vitro: role of parallel interneuronal processing. J Physiol (Lond) 483P: 191

[29] Gutierrez GJ, Grashow RG (2009) Cancer Borealis stomatogastric nervous system dissection. J Vis Exp: ([MedlinePgn:]) doi:10.3791/1207. PubMed: 19308017. PMC2705892 19308017

[30] Christie AE, Lundquist CT, Nässel DR, Nusbaum MP (1997) Two novel tachykinin-related peptides from the nervous system of the crab Cancer Borealis. J Exp Biol 200: 2279–2294. PubMed: 9316266. 9316266 10.1242/jeb.200.17.2279

[31] Goldberg D, Nusbaum MP, Marder EE (1988) Substance P-like immunoreactivity in the stomatogastric nervous systems of the crab Cancer borealis and the lobsters Panulirus interruptus and Homarus americanus. Cell Tissue Res 252: 515–522. doi:10.1007/BF00216638. PubMed: 2456155. 2456155

[32] Klagges BR, Heimbeck G, Godenschwege TA, Hofbauer A, Pflugfelder GO et al. (1996) Invertebrate synapsins: a single gene codes for several isoforms in Drosophila. J Neurosci 16: 3154–3165. PubMed: 8627354. 8627354 10.1523/JNEUROSCI.16-10-03154.1996 PMC6579133

[33] Skiebe P, Ganeshina O (2000) Synaptic neuropil in nerves of the crustacean stomatogastric nervous system: an immunocytochemical and electron microscopical study. J Comp Neurol 420: 373–397. doi:10.1002/(SICI)1096-9861(20000508)420:3. PubMed: 10754509. 10754509

[34] King DG (1976) Organization of crustacean neuropil. I. Patterns of synaptic connections in lobster stomatogastric ganglion. J Neurocytol 5: 207–237. doi:10.1007/BF01181657. PubMed: 1271087. 1271087

[35] Kilman VL, Marder E (1996) Ultrastructure of the stomatogastric ganglion neuropil of the crab, Cancer borealis. J Comp Neurol 374: 362–375. Available online at: doi:10.1002/(SICI)1096-9861(19961021)374:3&lt;362::AID-CNE5&gt;3.0.CO;2-# 8906505 10.1002/(SICI)1096-9861(19961021)374:3<362::AID-CNE5>3.0.CO;2-#

[36] Skiebe P, Wollenschläger T (2002) Putative neurohemal release zones in the stomatogastric nervous system of decapod crustaceans. J Comp Neurol 453: 280–291. doi:10.1002/cne.10398. PubMed: 12378588. 12378588

[37] Hedrich UBS, Smarandache CR, Stein W (2009) Differential activation of projection neurons by two sensory pathways contributes to motor pattern selection. J Neurophysiol 102: 2866–2879. doi:10.1152/jn.00618.2009. PubMed: 19741101. 19741101

[38] Stein W (2006) Functional consequences of activity-dependent synaptic enhancement at a crustacean neuromuscular junction. J Exp Biol 209: 1285–1300. doi:10.1242/jeb.02133. PubMed: 16547300. 16547300

[39] Coleman MJ, Nusbaum MP, Cournil I, Claiborne BJ (1992) Distribution of modulatory inputs to the stomatogastric ganglion of the crab, Cancer Borealis. J Comp Neurol 325: 581–594. doi:10.1002/cne.903250410. PubMed: 1361498. 1361498

[40] Nusbaum MP, Marder EE (1989) A modulatory proctolin-containing neuron (MPN). I. Identification and characterization. J Neurosci 9: 1591–1599. PubMed: 2566658. 2566658 10.1523/JNEUROSCI.09-05-01591.1989 PMC6569838

[41] Golowasch J, Marder EE (1992) Proctolin activates an inward current whose voltage dependence is modified by extracellular Ca2. J Neurosci 12: 810–817. PubMed: 1347561. 1347561 10.1523/JNEUROSCI.12-03-00810.1992 PMC6576042

[42] Blitz DM, Christie AE, Coleman MJ, Norris BJ, Marder E et al. (1999) Different proctolin neurons elicit distinct motor patterns from a multifunctional neuronal network. J Neurosci 19: 5449–5463. PubMed: 10377354. 10377354 10.1523/JNEUROSCI.19-13-05449.1999 PMC6782314

[43] Nusbaum MP, Blitz DM, Swensen AM, Wood D, Marder E (2001) The roles of co-transmission in neural network modulation. Trends Neurosci 24: 146–154. doi:10.1016/S0166-2236(00)01723-9. PubMed: 11182454. 11182454

[44] Coleman MJ, Nusbaum MP (1994) Functional consequences of compartmentalization of synaptic input. J Neurosci 14: 6544–6552. PubMed: 7965058. 7965058 10.1523/JNEUROSCI.14-11-06544.1994 PMC6577232

[45] Marder EE, Bucher D (2007) Understanding circuit dynamics using the stomatogastric nervous system of lobsters and crabs. Annu Rev Physiol 69: 291–316. doi:10.1146/annurev.physiol.69.031905.161516. PubMed: 17009928. 17009928

[46] Harris-Warrick RM, Marder EE (1991) Modulation of Neural Networks for Behavior. Annual. Reviews in the Neurosciences. 10.1146/annurev.ne.14.030191.000351 2031576

[47] Büschges A, Scholz H, Manira El A (2011) New Moves in Motor Control. Curr Biol 21: R513–R524. doi:10.1016/j.cub.2011.05.029. PubMed: 21741590. 21741590

[48] Marder EE, Goaillard J-M (2006) Variability, compensation and homeostasis in neuron and network function. Nat Rev Neurosci 7: 563–574. doi:10.1038/nrn1949. PubMed: 16791145. 16791145

[49] Goodman CS (1978) Isogenic grasshoppers: Genetic variability in the morphology of identified neurons. J Comp Neurol 182: 681–705. doi:10.1002/cne.901820408. PubMed: 721974. 721974

[50] Cabirol-Pol M-J, Combes D, Fénelon VS, Simmers J, Meyrand P (2002) Rare and spatially segregated release sites mediate a synaptic interaction between two identified network neurons. J Neurobiol 50: 150–163. doi:10.1002/neu.10023.abs. PubMed: 11793361. 11793361

[51] Baldwin DH, Graubard K (1995) Distribution of fine neurites of stomatogastric neurons of the crab Cancer borealis: evidence for a structured neuropil. J Comp Neurol 356: 355–367. doi:10.1002/cne.903560304. PubMed: 7642799. 7642799

